# Any overlap between orthorexia nervosa and obsessive–compulsive disorder in Lebanese adults? Results of a cross-sectional study and validation of the 12-item and 4-item obsessive–compulsive inventory (OCI-12 and OCI-4)

**DOI:** 10.1186/s12888-022-04119-3

**Published:** 2022-07-14

**Authors:** Souheil Hallit, Vanessa Azzi, Diana Malaeb, Sahar Obeid

**Affiliations:** 1 School of Medicine and Medical Sciences, Holy Spirit University of Kaslik, P.O. Box 446, Jounieh, Lebanon; 2 Psychology Department, College of Humanities, Effat University, Jeddah, 21478 Saudi Arabia; 3 Research Department, Psychiatric Hospital of the Cross, Jal Eddib, Lebanon; 4 School of Pharmacy, Gulf Medical University, Ajman, United Arab Emirates; 5 School of Pharmacy, Lebanese International University, Beirut, Lebanon; 6 Social and Education Sciences Department, School of Arts and Sciences, Lebanese American University, Jbeil, Lebanon

**Keywords:** Orthorexia nervosa, Eating disorders, Obsessive–compulsive disorder, Lebanon

## Abstract

**Background:**

Orthorexia Nervosa (ON), a compulsive obsession with vigorous eating, has increasingly caught researchers' attention. Although Orthorexia Nervosa has not been labeled an eating disorder, research about ON highlighted a strong link with anorexia nervosa or obsessive–compulsive disorder (OCD). Therefore, this study aimed to (1) validate the Arabic version of the Obsession-Compulsion Inventory (OCI-12 and OCI-4) and (2) check if there is an overlap between ON and OCD among a sample of Lebanese adults.

**Methods:**

This cross-sectional study involved 487 Lebanese participants between July and August 2021. The Dusseldorf Orthorexia Scale (DOS) was used to assess ON; scores between 25 and 29 indicate probable ON, whereas scores ≥ 30 indicate ON tendencies. A confirmatory factor analysis (CFA) was carried out using SPSS AMOS v.24 on the OCI-12 and OCI-4 scales’ items. The root mean square error of approximation (RMSEA) statistic, the Tucker Lewis Index (TLI) and the comparative fit index (CFI) were used to evaluate the goodness-of-fit of the model.

**Results:**

The CFA results indicated an excellent fit of the model: the Maximum Likelihood Chi-Square = 147.73 and Degrees of Freedom = 48, which gave a χ2/df = 3.08, TLI = 0.934, CFI = 0.952, and RMSEA = 0.065 [95% CI 0.054–0.078]. The fit indices of the one-factor structure of the OCI-4 were excellent as well: χ2/df = 6.15/2 = 3.08, TLI = 0.95, CFI = 0.98 and RMSEA = 0.065 [95% CI 0.007–0.127]. The Area Under the Curve was 0.600 [95% CI 0.524–0.674]. There was no cutoff value that showed good sensitivity or specificity at the same time. At the DOS cutoff of 25, sensitivity was 19.1%, whereas the specificity was 90.6%. The positive and negative predictive values (PPV and NPV) at this cutoff value were 24.4% and 88.7% respectively. At the DOS cutoff of 30, sensitivity was 8.8%, whereas the specificity was 94.3%. The PPV and NPV at this cutoff value were 10.6% and 92.5% respectively.

The results showed that higher total OCD scores (Beta = 0.15) were significantly associated with more ON tendencies. Moreover, higher OCD washing scores (Beta = 0.52), physical activity index (Beta = 0.06), and Body Mass Index (Beta = 0.17) were significantly associated with more ON tendencies.

**Conclusion:**

The present results suggest that ON, as measured by the DOS, shares more common features with disordered eating and cannot adequately predict the presence of OCD symptoms.

## Background

The term "orthorexia nervosa" (ON), a Greek term ("orthós" = right or correct and "orexsis" = hunger or appetite), designates an obsessive focus on following a vigorous eating pattern [1, 2]. Although ON is still not being classified in the Diagnostic and Statistical Manual of Mental Disorders (DSM-5) [3], current literature suppors the overlap between ON and anorexia nervosa [4] or obsessive–compulsive disorder (OCD) [5].

To this day, researchers have not agreed if ON should be considered a pathological entity on its own or if it should be considered as an adjunct to other mental disorders, most notably obsessive–compulsive [6]. On one hand, ON is deemed a separate clinical entity where affected individuals concentrate on the quality of consumed food which exerts a profound effect on daily lives and behaviors [7]. On the other hand, ON has a fundamental feature consisting of self-absorption into healthy eating, which coexists with an obsessive–compulsive personality [8].

The obsessive thinking in ON is typically related to food and calories, along with searching and finding necessary ingredients for preparing healthy meals [9, 10]. Fears about consuming contaminated or unsafe food to one's health and body are recurrent preoccupations, whereas regular examination and washing habits are stereotypical constraints in ON [11, 12]. The obsessive thoughts and habits about food are acceptable to individuals with ON [13] where those habits do not aim at reducing anxious symptoms in patients with OCD [14]. As a matter of fact, the persistence of food-related thoughts might imply an immoderate apprehension about preparing healthy food, rather than an irrational preoccupation followed by an impulse to neutralize it [15].

Recent research studies demonstrated an association between ON and physical activity [9, 10, 16–18]. The literature supports that orthorexia nervosa was associated with more aerobics and weight-training exercises in addition to higher levels of sports craving and impulsiveness [9]. Furthermore, previous literature supported the relationship between financial well-being and ON. Actually, individuals with high income have more financial ability to buy expensive high-quality food, a fundamental aspect of orthorexia nervosa, in addition to the further extensive information about nutritious food [19]. In addition, ON is linked with more inappropriate eating [19].

Multiple self-report scales exist to assess OCD symptoms, including but not limited to the Yale-Brown Obsessive Compulsive Scale self-report [20], Florida Obsessive–Compulsive Inventory [21], Dimensional Obsessive–Compulsive Scale [22] and Obsessive–Compulsive Inventory-Revised (OCI-R) [23]. Recently, the OCI-12 was created [24] as a modified shorter form of the OCI-R, which is composed of 18 items. The Arabic version of the OCI-12 is inexistent and not validated in Lebanon yet; a main advantage of validating this scale is that it yields 4 subscales (checking, ordering, washing and obsessing) compared to the other mentioned scales, giving a better understanding of OCD from different angles and perspectives. An even shorter version of the OCI-12 was created afterwards by the same authors [25].

The association between OCD and ON was tackled in few papers yet the results showed that those variables were not correlated with each other after adjustment over disordered eating symptoms [26, 27]. Although those results advocate that ON might be more related to disordered eating rather than OCD, the overlap remains theoretically between those two axes. Therefore, the objective of this study was to (1) validate the Arabic versions of the OCI-12 and OCI-4 scales and (2) check if there is overlap between ON and OCD among a sample of Lebanese adults.

## Methods

### Study design and participants

A total of 487 participants involved in this cross-sectional study (July–August 2021). Data was collected using the snowball technique; a Google form was designed and distributed via social media to persons from all Lebanese districts. The link of the study was circulated via social media where by the study participants were asked to forward that link to other people they know. Participants anonymity was obtained and all enrolled individuals had the freedom to accept or decline the invitation, with no monetary reward received in return.

### Minimal sample size calculation

The minimum required sample in this study was 395 participants according to the G-power system relying on an alpha error of 5%, a power of 80% and 10 confounding variables.

### Questionnaire

This survey was created in the native language of Lebanon (Arabic) and mandated around ten minutes to complete the form. It was divided into several sections:

#### Sociodemographic characteristics

This section gathered data about individuals' age, gender, educational level, marital status and Household Crowding Index (HCI). The HCI was obtained by dividing the number of persons living in the house by the number of rooms in the house (apart from the kitchen and bathrooms) [28]. Body Mass Index (BMI) was calculated by dividing weight (in Kg) by squared height (in meters). Physical activity Index was assessed by multiplying the daily activity intensity, frequency and duration [29].

#### Dusseldorf Orthorexia Scale (DOS)

It is a 10-item validated tool in Lebanon where higher scores indicate more tendencies towards ON behaviors [30, 31]. Scores between 25 and 29 indicate probable ON, whereas scores ≥ 30 reflect the presence of ON behaviors [32] (Cronbach’s alpha = 0.90).

#### The 12-item Obsession-Compulsion Inventory (OCI-12)

It is composed of 12 items scored on a 5-point Likert scale. Total scores ≥ 11 indicate the likely presence of OCD. This scale yields 4 subscales: checking, ordering, washing and obsessing [24]. The Cronbach’s alpha values were as follows: total scale (α = 0.87), checking (α = 0.71), ordering (α = 0.77), washing (α = 0.69) and obsessing (α = 0.81). The OCI-4 is a shorter form of the OCI-12, which considered one statement only from each dimension of the OCI-12 (α = 0.65).

#### Eating Attitude Test (EAT-26)

This tool is validated in Lebanon [33] and is composed of 26 questions. The responses for the questions are depicted through six options varying from infrequently to always. Scores ≥ 20 indicate disordered/inappropriate eating [34] (Cronbach’s alpha = 0.90).

### Statistical analysis

The study did not have missing values in the database since all questions were mandatory in the Google form and individuals were supposed to answer all questions before moving to the other sections. A confirmatory factor analysis (CFA) was carried out using SPSS AMOS v.24 on the OCI-12 and OCI-4 scales’ items, using the maximum-likelihood estimation. CFA was deemed preferred to the exploratory factor analysis since the factor structure of the scale is already known and we needed to test if it also fits for the Arabic version among Lebanese people. The root mean square error of approximation (RMSEA) statistic, the Tucker Lewis Index (TLI) and the comparative fit index (CFI) were used to evaluate the goodness-of-fit of the model as these are the most commonly used indices [35]. Values of RMSEA of 0.08 or less indicate a good-fitting model and values larger than 0.10 are indicative of a poor model [35]. On the other hand, TLI and CFI values greater than 0.95 indicate excellent model fit [35].

Receiver Operating Characteristics (ROC) curve analysis was conducted to check if ON measures are able to predict the presence/absence of OCD (tested variable: DOS score; state variable: presence of OCD symptoms); OCD symptoms were deemed present if the participant scores 11 or above on the OCI-12 scale [24]. We calculated the Area Under the Curve (AUC), sensitivity, specificity, positive and negative predictive values (PPV and NPV). The normality of distribution of the DOS score was verified (skewness and kurtosis values varying between -1 and + 1 [36]), in addition to a sample of more than 300 participants [37]. A linear regression was conducted, taking the DOS ON score as a dependent variable and all sociodemographic and OCD total and subscales scores as independent ones. *P* < 0.05 was deemed statistically significant. The SPSS software v.25 was used for all statistical analysis.

## Results

A total of 487 participants was enrolled in the study with a mean age of 28.38 ± 13.26 years and 67.2% females. Other characteristics and description of the scores can be found in Table 1.

**Table 1:** Sociodemographic characteristics of the participants (*N* = 487)

Variable	N (%)
**Gender**	
Female	372 (76.4%)
**Marital status**	
Single/divorced/widowed	392 (80.5%)
Married	95 (19.5%)
**Presence of OCD (yes)**	434 (89.1%)
**Presence of ON (at cutoff of 25) (yes)**	112 (23.0%)
**Presence of ON (at cutoff of 30) (yes)**	50 (10.3%)
	**Mean ± SD**
Age (in years)	24.41 ± 8.72
Body Mass index	23.20 ± 5.09
Household crowding index	1.64 ± 5.59
Physical activity index	22.67 ± 19.20
Financial wellbeing	4.71 ± 3.03
DOS ON total score	18.97 ± 7.47
OCD total score	22.42 ± 9.17
OCD checking	5.26 ± 2.79
OCD ordering	6.40 ± 2.93
OCD washing	5.28 ± 2.80
OCD obsessing	5.48 ± 3.25

### Confirmatory factor analyses of the OCI-12 and OCI-4

**Fig. 1:**
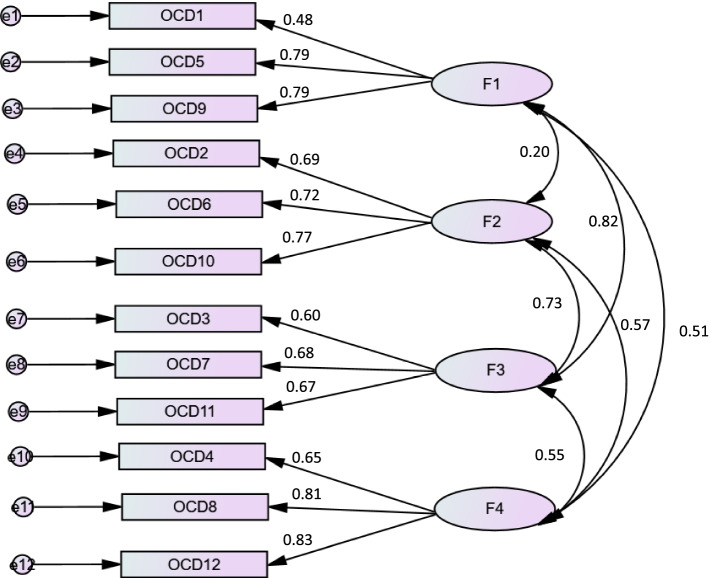
Standardized factor loadings of four-factor model of the Arabic version of the OCI-12 (*p* < 0.001 for all loading factors)

When validating the four-factor structure obtained in the original paper [24], the following results were obtained: the Maximum Likelihood Chi-Square = 147.73 and Degrees of Freedom = 48, which gave a χ2/df = 3.08. The TLI value was 0.93. The RMSEA value was 0.065 [95% CI 0.054–0.078] and CFI value was 0.95 respectively, indicating an excellent fit of the model (Fig. 1).

**Fig. 2:**
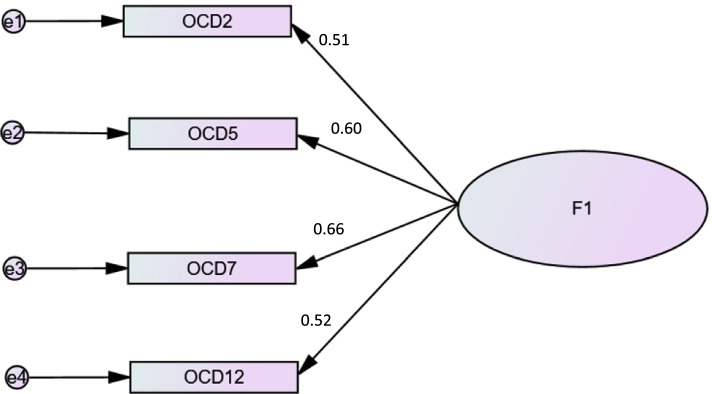
Standardized factor loadings of one-factor model of the Arabic version of the OCI-4 (*p* < 0.001 for all loading factors)

The fit indices of the one-factor structure of the OCI-4 were excellent as well: χ2/df = 6.15/2 = 3.08, TLI = 0.95, CFI = 0.98 and RMSEA = 0.065 [95% CI 0.007–0.127] (Fig. 2).

### ROC curve

**Fig. 3:**
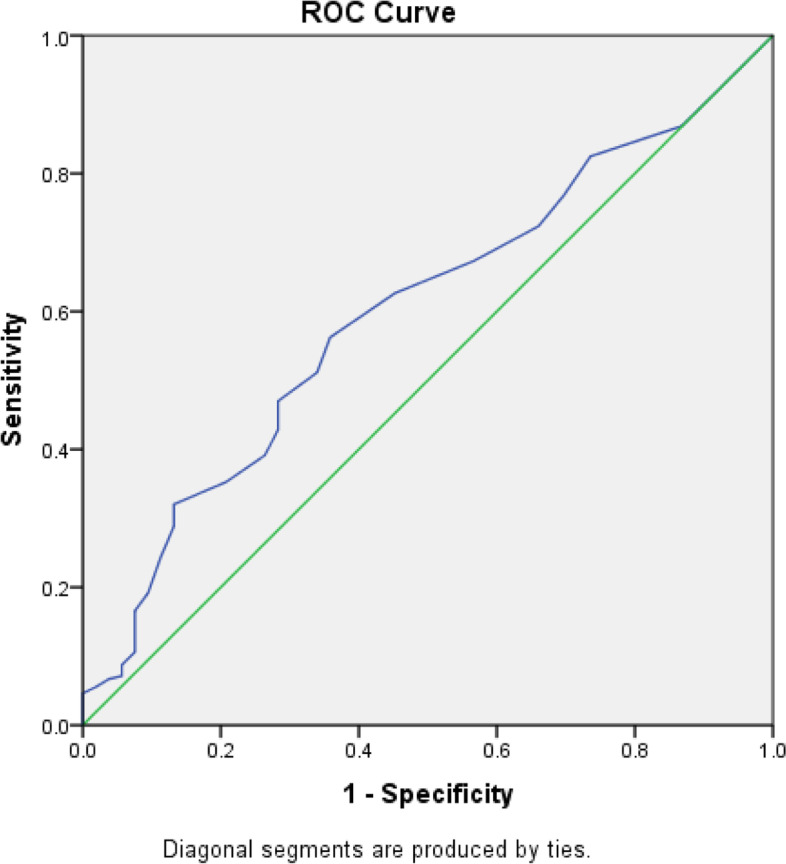
ROC curve of the DOS scale. Patients with OCD vs not (according to the OCI-12 scale) were analyzed. Area under the curve = 0.600 [95% CI 0.524–0.674] (*P* < 0.001); At DOS cutoff = 25: Se = 19.1% and Sp = 90.6%. At DOS cutoff = 30: Se = 8.8% and Sp = 94.3%

Patients with OCD vs not (according to the OCI-12 scale) were analyzed. The AUC was 0.600 [95% CI 0.524–0.674]. There was no cutoff value that showed a good sensitivity or specificity at the same time (Fig. 3). At the DOS cutoff of 25, sensitivity was 19.1%, whereas the specificity was 90.6%. The PPV and NPV at this cutoff value were 24.4% and 88.7% respectively. At the DOS cutoff of 30, sensitivity was 8.8%, whereas the specificity was 94.3%. The PPV and NPV at this cutoff value were 10.6% and 92.5% respectively.

**Fig. 4:**
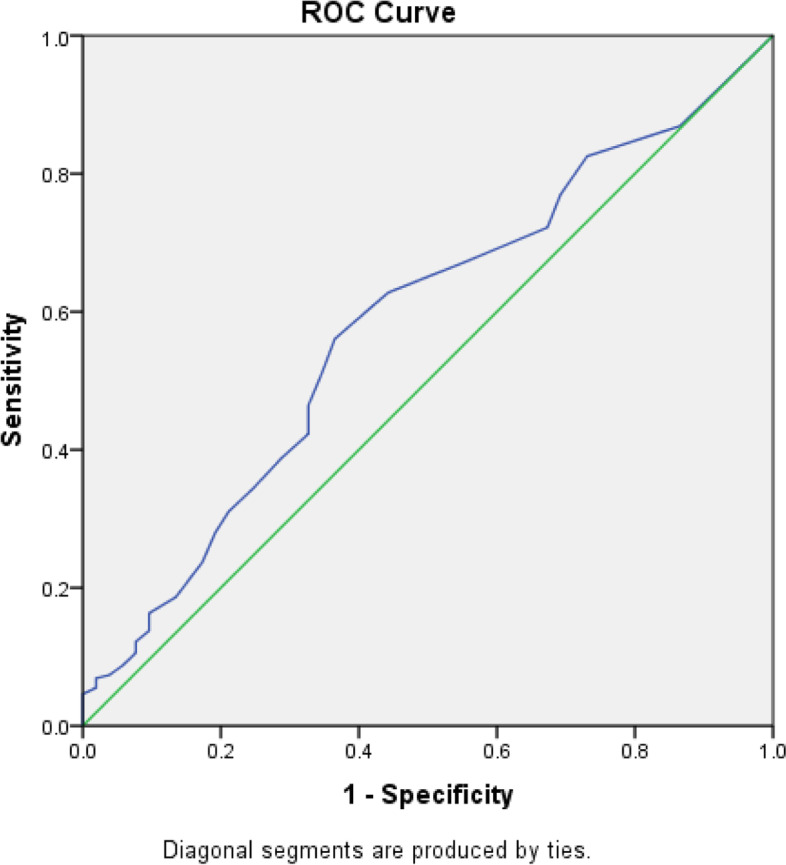
ROC curve of the DOS scale. Patients with OCD vs not (according to the OCI-12 scale) were analyzed. Area under the curve = 0.582 [95% CI 0.502–0.662] (*P* < 0.001); At DOS cutoff = 25: Se = 18.6% and Sp = 86.5%. At DOS cutoff = 30: Se = 8.7% and Sp = 94.2%

Similar results were obtained with the OCI-4 scale (Fig. 4).

### Multiple regression model

Higher total OCD scores (Beta = 0.14) were significantly associated with more ON tendencies (Table 2, Model 1). Moreover, higher OCD washing scores (Beta = 0.43), physical activity index (Beta = 0.07) and BMI (Beta = 0.16) were significantly associated with more ON tendencies (Table 2, Model 2).

**Table 2:** Multivariable analyses

**Variable**	**Unstandardized Beta**	**Standardized Beta**	***p***	**95% Confidence Interval**
**Model 1: Linear regression (using the ENTER method) taking the DOS total score as the dependent variable and OCD total score and sociodemographic variables as independent variables**				
OCD total score	0.14	0.18	** < 0.001**	0.07–0.21
Age	0.07	0.08	0.193	-0.04–0.17
Gender (females vs males*)	1.41	0.08	0.080	-0.17–3.00
Marital status (married vs single*)	-0.69	-0.04	0.543	-2.90–1.53
Financial wellbeing	0.06	0.02	0.599	-0.16–0.28
Body Mass Index	0.17	0.11	**0.020**	0.03–0.31
Household crowding index	-0.04	-0.03	0.475	-0.16–0.08
Physical activity index	0.08	0.19	** < 0.001**	0.04–0.11
**Model 2: Linear regression (using the ENTER method) taking the DOS total score as the dependent variable and OCD subscales and sociodemographic variables as independent variables**				
OCD checking	0.13	0.05	0.431	-0.19–0.44
OCD ordering	-0.01	-0.01	0.919	-0.29–0.26
OCD washing	0.43	0.16	**0.007**	0.12–0.75
OCD obsessing	0.03	0.02	0.775	-0.20–0.27
Age	0.06	0.08	0.225	-0.04–0.17
Gender (females vs males*)	1.58	0.09	0.052	-0.01–3.17
Marital status (married vs single*)	-0.68	-0.04	0.545	-2.90–1.53
Financial wellbeing	0.06	0.03	0.588	-0.16–0.28
Body Mass Index	0.16	0.11	**0.025**	0.02–0.30
Household crowding index	-0.04	-0.03	0.489	-0.16–0.08
Physical activity index	0.07	0.19	** < 0.001**	0.04–0.11
**Model 3: Linear regression (using the ENTER method) taking the DOS total score as the dependent variable and eating attitudes test, OCD subscales and sociodemographic variables as independent variables**				
EAT total score	0.13	0.24	** < 0.001**	0.08–0.18
OCD checking	0.14	0.05	0.370	-0.17–0.44
OCD ordering	0.05	0.02	0.701	-0.22–0.32
OCD washing	0.34	0.13	**0.032**	0.03–0.65
OCD obsessing	-0.02	-0.01	0.849	-0.25–0.21
Age	0.07	0.08	0.174	-0.03–0.17
Gender (females vs males*)	1.16	0.07	0.143	-0.39–2.72
Marital status (married vs single*)	-0.61	-0.03	0.578	-2.77–1.55
Financial wellbeing	0.11	0.05	0.313	-0.11–0.33
Body Mass Index	0.11	0.08	0.113	-0.03–0.25
Household crowding index	-0.10	-0.07	0.107	-0.21–0.02
Physical activity index	0.07	0.17	** < 0.001**	0.03–0.10

Numbers in bold indicate significant *p*-values; * indicates the reference group

When adding the EAT total score to the model, the results showed that higher EAT total scores (Beta = 0.13), OCD washing scores (Beta = 0.34), and physical activity index (Beta = 0.07) were significantly associated with more ON tendencies (Table 2, Model 3).

## Discussion

Our study findings showed that the DOS scale was poorly able to detect OCD at both cutoff points (25 and 30), with low PPV and high NPV values. The sensitivity values at both cutoff points were not adequate, while we obtained high specificity. The AUC value was < 0.7, indicating a poor ability of the DOS scale to predict OCD symptoms [38]. These results consolidate the findings of previous authors that ON and OCD are separate phenomena [39, 40] and that ON shares more common features with disordered eating rather than OCD [26, 27]. While there is no solid evidence about the correlation of ON with OCD, further research remain warranted to resolve this controversy. A review on psychosocial variables associated with ON [19] reported that perfectionism, orthorexia traits, psychopathology, disordered eating and antecedants, dieting, negative body image and drive for thinness were associated with higher ON. Barthels et al. [41] suggested that ON and ED share common psychopathological characteristics such as low body acceptance, which links both disorders together.

The results in the regression analyses highlighted that higher OCD and EAT total scores (more inappropriate eating) were significantly associated with more ON tendencies and behaviors. As mentioned in the introduction, ON is characterized by obsessions and compulsions about the quality of food [42]. Previous studies concluded that more OCD symptoms are linked with more ON behaviors [2, 43–45]. In particular, the OCI washing subscale was found to be significantly associated with ON; we hypothesize that this finding might be related to the fact that during the COVID-19 pandemic, handwashing is applied as an essential measure to prevent the disease’s spread [46, 47], since hands are a known vector in the transmission of microorganisms. During the pandemic, there has been extensive efforts implemented to raise awareness towards handwashing as recommended by the World Health Organization and other healthcare institutions to stop the COVID-19 virus spread. Our findings showed a close association between EAT and ON, which can be demonstrated by the fact that EAT assesses disorderd eating habits together with the social pressure, food pre- occupation, purging behaviors and food awareness, which are also able to predict orthorexic behaviors [48]. Furthermore, ON share similarity with eating disorders where both have impulsive persistent healthy food thoughts.

Higher physical activity index was significantly associated with more ON, consolidating the findings of previous papers [18, 43] that found a significant close relation between ON symptomatology and physical activity. A recent systematic review reinforced this relationship and showed a slight correlation with exercise and moderately with exercise addiction, conveying a shared variance between those two conducts [49]. Our results can be explained by the fact that orthorexic people strictly exercise to promote their health as engagement in regular physical activity plays a vital role in a healthy lifestyle, weight and stress management [50].

### Clinical implications

Studying the relation between psychological disorders and ON has a potential advantage to highlight treatment recommendations and preventive guidelines applications. In addition, understanding the impact of personality traits along with eating disorders on ON individuals improves prediction about the risk of ON manifestations. Identification of eating disorders features by enhancing patient education about the various etiologic factors (physical activity, perception of food quality and quantity) can be translated into decreased progression of ON.

### Limitations

This is a cross-sectional study where cause-effect relationship and causality of the relationship between OCD and ON cannot be established. The study took into account many factors associated with ON but other factors might not be taken into consideration, which introduced the risk of residual confounding. Symptoms were self-reported and not evaluated by a clinician. Data was collected online, excluding those who do not have Internet access or a smartphone, predisposing us to a selection bias. The majority of the participants was females and with a low mean age, thus, the results cannot be generalized to the whole population. Information bias is also present since participants might give wrong information in all cross-sectional studies. The design is limited due to the use of self-report assessment (and not a psychiatrist diagnosis), internet-based tools to validate an instrument and the lack of a control group. Finally, there is no valid and coherent gold standard measure to define which participants are classified as ON and which are not.

## Conclusion

A better comprehension of the different psychological disorders in ON establishes opportunities to prevent and intervene in these cases. Overall, the current study identified that ON, as measured by the DOS, cannot adequately predict the presence of OCD symptoms. Results suggest classifying ON possibly as an eating disorder rather than being related to OCD. Furthermore, future research that considers the different psychological disorders and the trigger factors of their development including eating disorders, can contribute to the understanding of the association between ON and OCD.

## Data Availability

The authors do not have the right to share any data information as per their institutions policies. The dataset supporting the conclusions is available upon request to the corresponding author.

## Abbreviations

ON: Orthorexia Nervosa
OCD: Obsessive–compulsive disorder
OCI-12: Obsession-Compulsion Inventory
DOS: Dusseldorf Orthorexia Scale
CFA: Confirmatory factor analysis
RMSEA: Root mean square error of approximation
TLI: Tucker Lewis Index
CFI: Comparative fit index
PPV: Positive predictive value
NPV: Negative predictive value
